# Exogenous Urokinase Inhibits Proteasomal Degradation of Its Cognate Urokinase Plasminogen Activator Receptor

**DOI:** 10.3389/fphar.2022.754271

**Published:** 2022-08-12

**Authors:** Ran Zhu, Ting-Wei Liu, Fan Liu

**Affiliations:** ^1^ Department of Critical Care Medicine, The First Hospital of China Medical University, Shenyang, China; ^2^ Department of Respiratory and Critical Care Medicine, The First Hospital of China Medical University, Shenyang, China

**Keywords:** acute pulmonary embolism, hypoxia/reoxygenation, urokinase-like plasminogen activator, uPA, uPAR, ubiquitylation

## Abstract

Acute pulmonary embolism (APE) is a debilitating condition with high incidence and mortality rates. APE is widely treated with the serine protease urokinase or urokinase-type plasminogen activator (uPA) that functions by resolving blood clots *via* catalyzing the conversion of plasminogen to plasmin. Treatment with recombinant uPA has been shown to increase endogenous expression of uPA and its cognate receptor, uPAR; however, the mechanisms for this induction are not known. Using an *in vitro* hypoxia/reoxygenation model in bronchial epithelial BEAS-2B cells, we show that induction of hypoxia/reoxygenation induces apoptosis and increases secretion of tumor necrosis factor–alpha, brain natriuretic peptide, and fractalkine, which are attenuated when treated with exogenous uPA. Induction of hypoxia/reoxygenation resulted in decreased expression of uPAR on cell surface without any significant changes in its messenger RNA expression, highlighting post-transcriptional regulatory mechanisms. Determination of uPAR protein half-life using cycloheximide showed treatment with uPA significantly increased its half-life (209.6 ± 0.2 min from 48.2 ± 2.3 min). Hypoxia/reoxygenation promoted the degradation of uPAR. Inhibition of proteasome-mediated degradation using MG-132 and lactacystin revealed that uPAR was actively degraded when hypoxia/reoxygenation was induced and that it was reversed when treated with exogenous uPA. Determination of the proteolytic activity of 20S proteasome showed a global increase in ubiquitin–proteasome activation without an increase in proteasome content in cells subjected to hypoxia/reoxygenation. Our results cumulatively reveal that uPAR is actively degraded following hypoxia/reoxygenation, and the degradation was significantly weakened by exogenous uPA treatment. Given the importance of the uPA/uPAR axis in a multitude of pathophysiological contexts, these findings provide important yet undefined mechanistic insights.

## Introduction

Blood clot-mediated blockage of the pulmonary artery resulting in obstruction of pulmonary circulation and hemodynamic collapse is termed as acute pulmonary embolism (APE) (Cohen et al., 2007; Geerts et al., 2008; Lang et al., 2013). Due to the paucity of symptoms and the absence of an appropriate diagnosis, the exact epidemiological details of APE are unknown. A multicenter study in China between 1997 and 2008 estimated that the incidence rate of APE is 0.1% with a higher incidence in males than in females (Yang et al., 2011). Given the advancement of treatment protocols, the mortality rate significantly decreased over the years—from 25.1% in 1997 to 8.1% in 2008 (Yang et al., 2011). However, it remains a seriously debilitating condition for which optimal therapies are required.

It has been well documented that APE is associated with inflammatory response and cell death, in parts mediated by mitogen-activated protein kinase, phosphoinositide 3-kinases/protein kinase B, and nuclear factor–kappa beta signaling pathways (Apostolakis and Spandidos, 2013; Wang et al., 2013; Wang et al., 2014). APE-associated ischemia and pulmonary hypertension induce an increase in serum levels of cytokines and chemokines, including tumor necrosis factor–alpha (TNFα), interleukin (IL)-1β, IL-8, CX3CR1, CXCRL1, brain natriuretic peptide (BNP), troponin T, and D-dimer (Zagorski et al., 2003; Wang et al., 2013; Wang et al., 2014; Zhang et al., 2017; Shi et al., 2018). Pro-inflammatory chemokines and cytokines induce infiltration of immune cells in the lungs, including natural killer cells and T cells (Lang et al., 2013; Saghazadeh et al., 2015; Saghazadeh and Rezaei, 2016).

Urokinase or urokinase (UK)-type plasminogen activator (uPA), a serine protease that functions by resolving clots *via* catalyzing the conversion of plasminogen to plasmin, is the most widely used drug for treating APE (Sasahara et al., 1967; Cheng et al., 2002). Indeed, dose–effect and duration–effect clinical trials have been performed to study the outcome of UK treatment in patients with APE (Zhang et al., 2007; Wang et al., 2009; Zhao et al., 2018; Zhang et al., 2019).

Administration of pro-UK or exogenous uPA increases the expression of endogenous u-PA in circulating blood, lung epithelial cells, and mononuclear cells in animal models or patients with APE (Sasaki et al., 1985; Sumi et al., 1985; Toki et al., 1985). uPA mediates its activity by binding to its high-affinity glycosyl phosphatidylinositol (GPI)-linked protein receptor called UK plasminogen activator receptor (uPAR) (Ploug et al., 1991; Leth et al., 2019; Xu et al., 2020). Indeed, exogenous uPA has been shown to induce the expression of uPAR (Toki et al., 1985). Interaction of uPA with uPAR is critical for its activity in resolving APE (Bdeir et al., 2000; Liu et al., 2008).

However, how exogenous UK induces uPAR expression is not defined. Understanding the same will provide critical information that might be utilized in developing additional therapeutic regimens or optimizing current ones for patients with APE. Hence, using an *in vitro* model of APE (hypoxia/reoxygenation), we determined the mechanism underlying the increase in uPAR expression following treatment with exogenous UK. Our results show that uPAR is ubiquitinated at lysine 48 residues and actively degraded in APE, whereas the addition of exogenous UK inhibits ubiquitination and degradation of uPAR.

## Materials and Methods

### *In Vitro* Hypoxia/Reoxygenation Model and Treatments

Human bronchial epithelial cells (BEAS-2B) (ATCC) were cultured in Dulbecco's modified eagle medium (DMEM) containing 5% FBS, 100 U/mL penicillin, and 100 μg/ml streptomycin in 5% CO_2_ at 37°C. To establish the hypoxia/reoxygenation (H/R) model, BEAS-2B cells were incubated in hypoxic conditions (1% O_2_, 5% CO_2_, and 94% N_2_) in serum and glucose-free DMEM for 12 h. Postincubation under hypoxic conditions, the medium was changed to a normal growth medium and cells were incubated for an additional 12 h under normoxic conditions (5% CO_2_), where indicated cells were treated with recombinant UK (10 ng/ml (American Diagnostica, Stamford, CT)). Cells maintained throughout under normoxic conditions were used as controls. For phospholipase C (PLC) treatment, BEAS-2B cells were treated with phosphatidylinositol-specific PLC (PI-PLC; 0.5 IU/ml) (Sigma Aldrich) for 2 h at 37°C before induction of H/R.

### Terminal Deoxynucleotidyl Transferase Biotin-dUTP Nick End Labeling Assay

BEAS-2B cells under normoxic conditions, H/R conditions, and cells that were treated with UK were all labeled using the terminal deoxynucleotidyl transferase biotin-dUTP nick end labeling (TUNEL) assay kit (R&D Systems) according to the manufacturer’s instructions. TUNEL-labeled cells were counterstained with 4′,6-diamidino-2-phenylindole (DAPI) and subsequently washed thrice with phosphate-buffered saline (PBS). Apoptosis rate was defined as (number of apoptotic cells/total number of cells) × 100%.

### Determination of Cytokines

BEAS-2B cells under normoxic conditions, H/R conditions, and cells that were treated with UK were pelleted by centrifuging at 1,000 g for 5 min at 4°C. The supernatant was used to determine the expression of indicated cytokines. Luminex (Millipore) was used to quantify the levels of the cytokines.

### RNA Isolation and Real-Time Quantitative Polymerase Chain Reaction

Total RNA was extracted using TRIzol (Thermo Fisher Scientific). SuperScript III reverse transcriptase (Thermo Fisher Scientific) was used for cDNA synthesis. Real-time quantitative polymerase chain reaction (RT-qPCR) reactions were set up using KAPA SYBR FAST (KAPA BIOSYSTEMS, Wilmington, United States). The primers used were: *PLAUR*—forward primer: 5′-CCA​CTC​AGA​GAA​GAC​CAA​CAG​G-3′; reverse primer: 5′-GTA​ACG​GCT​TCG​GGA​ATA​GGT​G-3′; *PLAU*—forward primer: 5′- GGC​TTA​ACT​CCA​ACA​CGC​AAG​G-3′; reverse primer: 5′-CCT​CCT​TGG​AAC​GGA​TCT​TCA​G-3′. Raw Ct values were normalized to the glyceraldehyde-3-phosphate dehydrogenase (*GAPDH*) gene (forward primer: 5′- GTC​TCC​TCT​GAC​TTC​AAC​AGC​G-3′; reverse primer: 5′-ACC​ACC​CTG​TTG​CTG​TAG​CCA​A-3′), and changes in expression were calculated using the 2^−ΔΔCt^ method.

### Immunofluorescence

BEAS-2B cells in each group were fixed using 4% polyformaldehyde for 30 min at room temperature (24°C). Cells were permeabilized using 0.1% Triton X-100 for 5 min at room temperature. Cells were subsequently blocked using 10% bovine serum albumin for 1 h at room temperature before incubation with anti-uPA (SC-59727, Santa Cruz, 1:125 dilution) and anti-uPAR (SC-10815, Santa Cruz, 1:200 dilution) overnight at 4°C. Cells were washed thrice using PBS and then incubated with antirabbit immunoglobulin G (IgG) for 1 h at 37°C and washed again thrice with PBS. Cell slides were finally stained with DAPI and mounted with an antifade mounting medium and imaged *via* confocal microscopy.

### Cycloheximide and MG-132 Treatment

BEAS-2B cells subjected to H/R ± UK were treated with 10 μg/ml cycloheximide (CHX) (Sigma Aldrich) for up to 4 h after H/R (12 h hypoxia and 12 h reoxygenation). Post-treatment cells were washed with ice-cold PBS containing CHX and then processed for western blotting as described below. The relative amount of uPAR protein left after each time point was used to calculate the half-life of uPAR in BEAS-2B cells ± UK. To inhibit proteasomal degradation, BEAS-2B cells subjected to H/R ± UK were treated with 20 μM of MG-132 (Sigma Aldrich) for up to 2 h. At the end of each time point, cells were harvested, washed with ice-cold PBS, and then processed for western blotting as described below.

### Western Blotting

The cells mentioned above were washed with ice-cold PBS and then lysed using radioimmunoprecipitation assay lysis buffer (ThermoFisher Scientific). BCA protein assay kit (ThermoFisher Scientific) was used to determine protein concentrations. Proteins were run on SDS-PAGE gels and transferred to polyvinylidene fluoride membranes. Antibodies used to probe the blots were purchased from Cell Signaling, Cambridge, United States, and used at 1:1000 dilution; incubation with primary antibody was overnight at 4°C. Blots were probed with GAPDH to confirm equal loading of protein across the different samples. Densitometry analysis was performed using NIH Image J software (https://imagej.nih.gov/ij/; accessed on 10 November 2020).

### Isolation of 20S Proteasomes

Hobler et al. (1999)’s protocol was followed for isolation of 20S proteasome and determination of proteolytic activity. BEAS-2B cell pellets were resuspended in buffer (pH 7.5) containing 50 mM Tris-HCl, 250 mM sucrose, 5 mM MgCl_2_, and 1 mM dithiothreitol (DTT) and supplemented with protease and phosphatase inhibitor cocktail (ThermoFisher Scientific). The suspension was subjected to three rounds of centrifugation: 10,000 g for 20 min, 100,000 g for 1 h, and 100,000 g for 5 h—all at 4°C, with each subsequent centrifugation performed with the supernatant from the prior centrifugation step. The final pellet was resuspended in a buffer containing 50 mM Tris-HCl, 5 mM MgCl_2_, and 20% (v/v) glycerol. Protein content was determined using the BCA assay as described above.

### Determination of Proteolytic Activity

Fluorogenic peptides N-carbenzoxy-Leu-Leu-Glu-7-amido-4-methylcoumarin (LLE) and succinyl-Leu-Leu-Val-Tyr-7-amido-4-methylcoumarin (LLVY) were purchased from Sigma Aldrich. Proteolytic activity of 20S proteasome was determined in terms of chymotrypsin-like and peptidylglutamyl peptide-hydrolyzing peptidase-mediated hydrolysis of the fluorogenic substrates (Craiu et al., 1997). Reactions containing 15 μg 20S proteasome extract in 60 μl of buffer (62.5 mM Tris-HCl, 12.5 mM MgCl_2_, 1.2 mM DTT, 0.01 U apyrase, and either 375 μM of LLE or 100 μM of LLVY) were incubated for 45 min at 37°C. Proteolytic activity (pM per μg protein per min) was determined by assaying for the methylcoumarylaide cleavage product (380 nM excitation and 440 nM emission) (Craiu et al., 1997).

### Immunoprecipitation

Protein lysates (150 μg) from BEAS-2B cells subjected with or without the H/R (without UK) ± MG-132 (for either 2 h or indicated time points) were immunoprecipitated using 5 μg of anti-uPAR antibody (cell signaling, #12863)/normal rabbit IgG (cell signaling, #2729) and Pierce Classic Magnetic IP/Co-IP kit (ThermoFisher Scientific). Successful immunoprecipitation was confirmed *via* western blotting using an anti-uPAR antibody. The primary antibodies were as follows: K48-linkage-specific polyubiquitin antibody (cell signaling, #4289) and K63-linkage-specific polyubiquitin antibody (cell signaling, #5621); Anti-Ub (Santa Cruz Biotechnology, sc166553).

### Statistical Analysis

Data were expressed as mean ± standard deviation (SD). Statistical significance was determined using a one-way analysis of variance followed by Tukey’s post hoc test for multiple comparisons; *p* < 0.05 was considered statistically significant.

## Results

**FIGURE 1:**
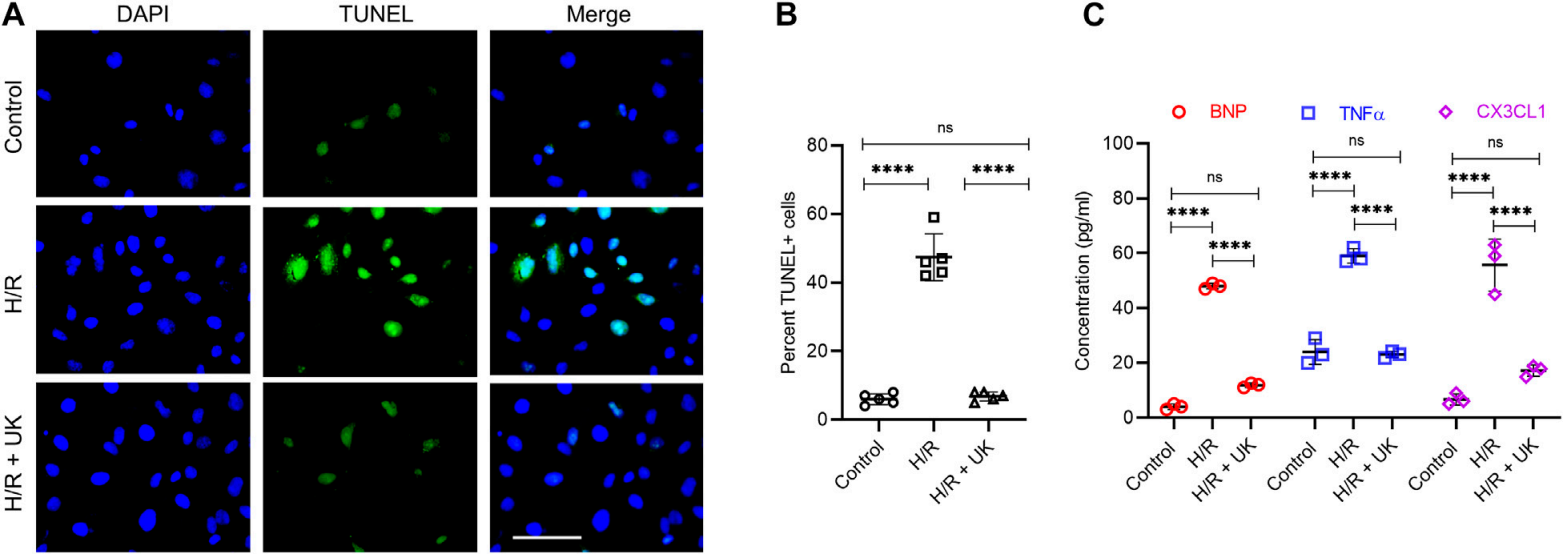
Urokinase inhibits H/R-induced cell death and cytokine secretion. An *in vitro* model of APE in BEAS-2B cells. Cells were subjected to hypoxic conditions for 12 h followed by reoxygenation for 12 h (for details refer to *Materials and Methods*). **(A)** Representative images from terminal deoxynucleotidyl transferase biotin-dUTP nick end labeling assay. **(B)** Graph shows quantification of images in **(A)**. Data are represented as mean ± SD; ****p < 0.0001, *ns*, not significant (*n* = 5). **(C)** Secretion of indicated cytokines in the cell supernatant was determined using Luminex assay. represented as mean ± SD; ****p < 0.0001, ns, not significant (*n* = 3) (UK: urokinase).

To investigate the mechanism of action of exogenously added uPA, we used an *in vitro* H/R model in which BEAS-2B cells were exposed to hypoxic conditions for 12 h followed by reoxygenation under normoxic conditions for 12 h. Compared to control cells maintained throughout under normoxic conditions, H/R significantly induced cell death as assessed *via* TUNEL staining (Figures 1A,B). Treatment with UK significantly decreased apoptosis induced by H/R to levels observed in control cells (Figures 1A,B). Since APE is associated with increased secretion of inflammatory cytokines (Zagorski et al., 2003; Wang et al., 2013; Wang et al., 2014; Zhang et al., 2017; Shi et al., 2018), we next determined the expression of BNP, TNFα, and fractalkine (CX3CL1) in cell supernatants. Compared to control cells, H/R resulted in a significant increase in the expression of BNP, TNFα, and CX3CL1 (Figure 1C). Treatment with UK significantly decreased the secretion of these cytokines in cell supernatants to levels comparable to that observed in control cells (Figure 1C). These results confirmed that the H/R model in BEAS-2B cells can be implemented as a system to define the mechanism of action of exogenous uPA.

**FIGURE 2:**
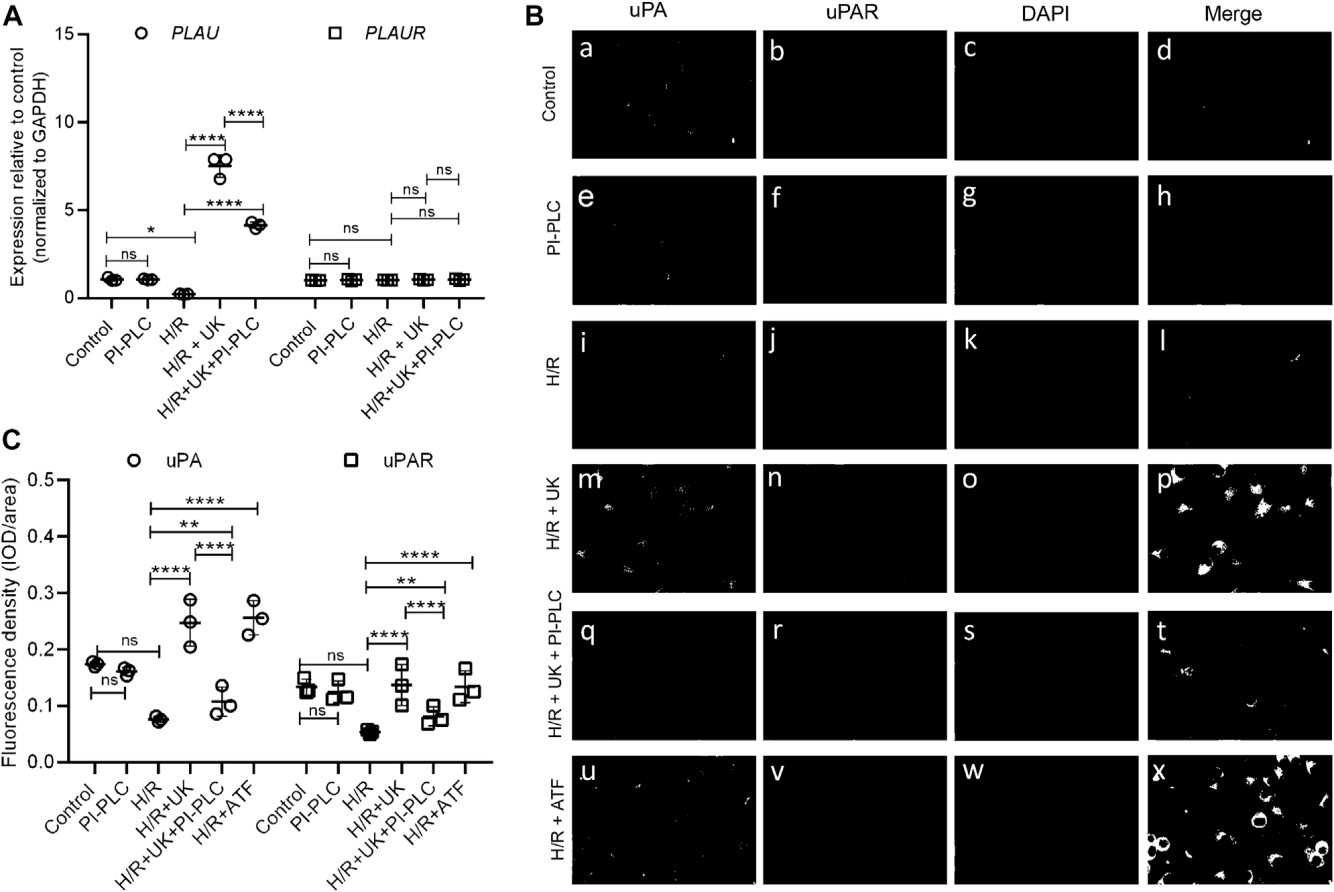
Exogenous uPA treatment induces uPAR protein expression without changes in its transcript expression. **(A)** Relative expression of *PLAU* (encoding uPA) and *PLAUR* (encoding uPAR) was determined in indicated conditions using RT-qPCR. Data are represented as mean ± SD; **p < 0.05, ****p < 0.0001, ns, not significant (*n* = 3). **(B)** Representative immunofluorescence images of uPA and uPAR expression in BEAS-2B cells, including control group (a–d) and cells subjected to PI-PLC (e–h), H/R (i–l), H/R + UK (m–p), and H/R + UK but pretreated with PI-PLC (q–t). Scale bar, 100 μm. **(C)** Relative expression of uPA and uPAR. The relative expression was based on the fluorescence intensity of IF and the signal was quantified using Image-Pro Plus (UK: urokinase).

Given that it was earlier shown that exogenous pro-UK can increase the expression of both endogenous uPA and its cognate receptor uPAR (Liu et al., 2008), we next determined the mRNA expression of *PLAU* (encoding uPA) and *PLAUR* (encoding uPAR) in the BEAS-2B cells. Compared to control condition, the expression of *PLAU* significantly decreased following H/R (Figure 2A), which was restored when treated with UK (Figure 2A). However, there was no significant difference in the expression of *PLAUR* in BEAS-2B cells grown under control conditions or when subjected to H/R ± UK (Figure 2A). We next performed immunofluorescence analysis of uPA and uPAR expression on BEAS-2B cells. Compared to control condition, the expression of uPA and uPAR significantly decreased following H/R (Figures 2B,C). However, treatment with UK resulted in a robust increase in expression of both uPA and uPAR compared to H/R condition (Figures 2B,C). To determine if the increase in uPAR was due to uPA, we pretreated the BEAS-2B cells with PI-PLC. Given that uPAR is a GPI-linked protein, it will be removed from the cell surface when treated with PI-PLC (Figures 2B,C). This also resulted in decreased expression of uPA compared to H/R + UK condition (Figures 2B,C), confirming that the binding of exogenous uPA to uPAR is involved in the endogenous increase in uPA expression. Considering that the amino-terminal fragment of uPA (ATF) can bind and activate uPAR but lacks catalytic activity, we treated the BEAS-2B cells following H/R with ATF, which has the same effect as treatment with UK (Figures 2B,C). To determine whether the pretreatment of PI-PLC changes the expression of uPAR on mRNA level as the duration of PI-PLC treatment is fairly long, 2 h + 24 h, we did an RT-qPCR of uPAR of BEAS-2B cells pretreated with PI-PLC. As shown in Figure 2A, PI-PLC treatment almost has no influence on the expression of uPAR on the mRNA level. This was also not due to toxic effect of PI-PLC as cells treated with this reagent showed similar cell growth kinetics as untreated cells. These results also indicated that the observed increase in uPAR expression following treatment with UK was being regulated at the post-transcriptional level.

Post-transcriptional gene regulation can be mediated by either translational inhibition of the mRNA or post-translational modifications of a newly synthesized protein that results in altered stability. Translational inhibition is normally mediated by microRNA, which binds to its target mRNA and causes its degradation. Since we did not observe any significant change in *PLAUR* expression under the different conditions (Figure 2A), we hypothesized that it is being regulated by post-translational modification.

**FIGURE 3:**
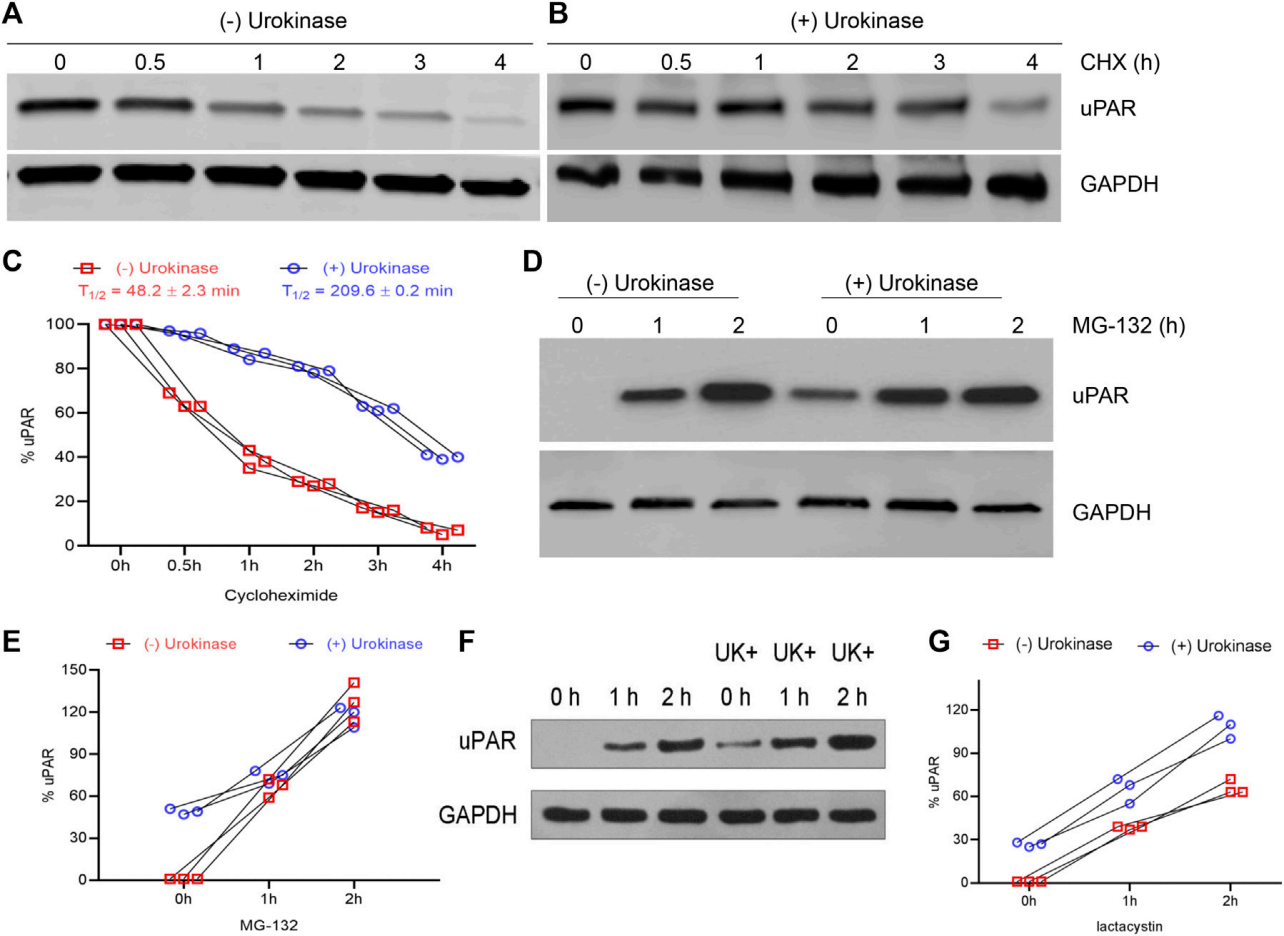
H/R induces degradation of uPAR protein, which can be inhibited using UK. **(A–C)** BEAS-2B cells subjected to H/R ± UK were treated with 10 μg/ml cycloheximide for indicated times and lysates were probed with anti-uPAR and GAPDH antibodies. Shown are representative blots **(A,B)** and quantification of protein half-life **(C)**. **(D,E)** Same as **(A,B)** but treated with 20 μM of the proteasome inhibitor MG-132 **(D)** and quantification of protein half-life **(E)**. **(F,G)** Same as **(A)** and **(B)** but treated with 20 μM of the proteasome inhibitor lactacystin **(F)** and quantification of protein half-life **(G)** (UK: urokinase).

Hence, we next determined the half-life of uPAR in BEAS-2B cells subjected to H/R ± UK. At the end of H/R (24 h), cells were treated with cycloheximide for up to 4 h. Cycloheximide binds to the 60S ribosome subunit and inhibits translation by preventing translational elongation. Lysates from cells harvested from cells subjected to different time points of cycloheximide treatment were probed with an anti-uPAR antibody. In BEAS-2B cells subjected to H/R, uPAR protein degraded faster (Figure 3A) compared to that treated with UK (Figure 3B). Densitometry analysis using NIH Image J was used to calculate the half-life of uPAR under the H/R ± UK conditions. Urokinase treatment significantly increased the half-life of uPAR to 209.6 ± 0.2 min from 48.2 ± 2.3 min (cells without UK treatment) (Figure 3C). These results indicated that uPAR protein is being actively degraded following H/R and the degradation is weakened when treated with exogenous uPA. To confirm that we next treated the cells with the proteasome inhibitor MG-132 for up to 2 h following the 24 h H/R regimen ± UK. Treatment with MG-132 resulted in a robust increase in uPAR protein expression in BEAS-2B cells even without UK treatment, confirming that uPAR protein is actively degraded when exposed to H/R. Treatment with MG-132 also resulted in a further increase in uPAR protein in the cells treated with UK (Figures 3D,E). To further confirm the results above, lactacystin, an inhibitor specific to the proteasome, was also used to treat the cells for up to 2 h following the 24 h H/R regimen ± UK; the result was shown in Figures 3F,G. These results cumulatively indicated that uPAR protein is degraded when subjected to H/R, but the degradation is weakened following treatment with exogenous uPA.

**FIGURE 4:**
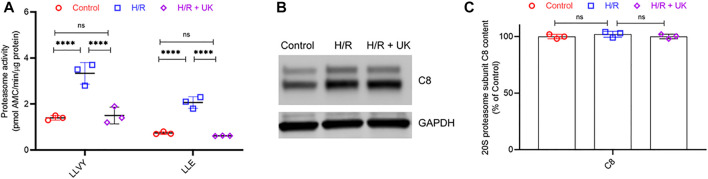
H/R induces global activation of the proteolytic activity of proteasome without induction of proteasome content. **(A)** Proteasome activity in BEAS-2B cells under control conditions or following H/R ± urokinase (UK). Proteasome activity against fluorogenic LLE and LLVY substrates was significantly higher following HR compared to control. Urokinase treatment significantly decreased the proteolytic activity. Data are represented as mean ± SD; ****p < 0.0001, ns, not significant (*n* = 3). *AMC*. amido-4-methylcoumarin. **(B,C)** 20S proteasome subunit C8 protein content in BEAS-2B cells under control conditions or following H/R ± UK. Shown are representative blots **(B)** and quantification **(C)**. Data in ***C*** are represented as mean ± SD; ns, not significant (*n* = 3).

To understand if H/R resulted in global activation of proteasome activity in the BEAS-2B cells or was specific to uPAR, we next determined the proteolytic activity of 20S proteasome in the BEAS-2B cells maintained under control conditions or subjected to HR ± UK. 20S proteasomes were isolated from the cells of different experimental groups and their proteolytic activity against two fluorogenic substrates chymotrypsin-like (LLVY) and peptidylglutamyl peptide-hydrolyzing (LLE) peptidase was quantified. Proteasome activity against both substrates was significantly higher in cells subjected to H/R compared to that of the control group (Figure 4A). Treatment with UK resulted in significant inhibition of both the LLVY and LLE peptidase activities of the proteasome (Figure 4A). An increase in proteolytic activity can either be due to an increase in specific activity of the proteasome or an increase in the proteasome content. Hence, we next assessed protein expression of the C8 subunit of the 20S proteasome. There was no significant difference in the C8 protein expression between control and H/R ± UK conditions (Figures 4B,C), confirming that the increased proteolytic activity of the proteasome post-H/R was due to not an increased number of proteasomes but increased activity of the proteasomes. Taken together, these results established that H/R results in global activation of proteasomal activity, which can be reversed by treatment with exogenous uPA.

**FIGURE 5:**
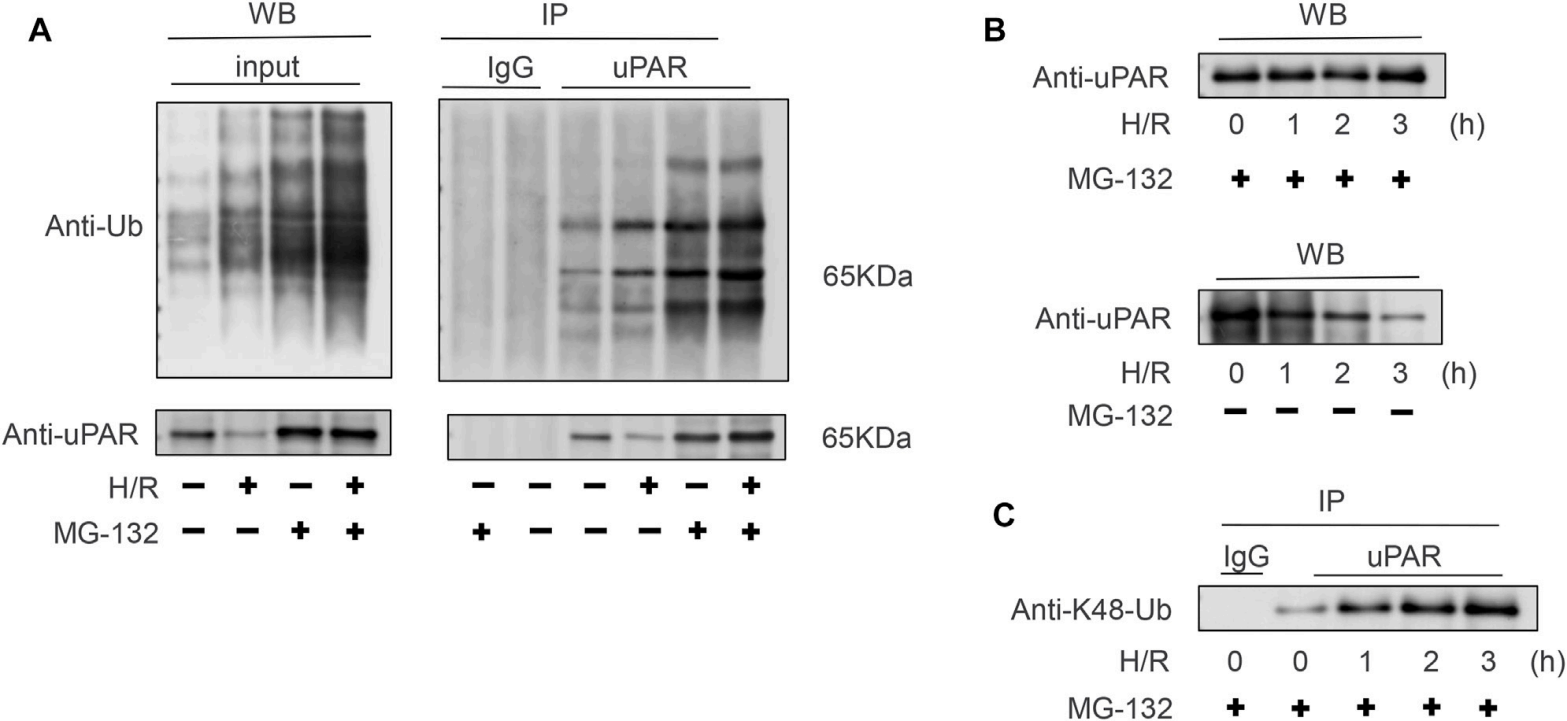
Polyubiquitination of uPAR occurs *via* lysine 48-linked chains. **(A)** Lysates obtained from BEAS-2B cells subjected with or without H/R ± MG-132 were immunoprecipitated using an anti-uPAR antibody. To confirm successful ubiquitination, blots were probed with an anti-Ub antibody to determine poly-ubiquitination and anti-uPAR antibodies. **(B)** BEAS-2B cells subjected to H/R for indicated time points underwent MG-132; western blotting was done with anti-uPAR. **(C)** Same as **(B)**, but the lysates were immunoprecipitated using anti-uPAR and detected with anti-lysine 48K-Ub. Shown are representative blots from three independent experiments. The results showed that uPAR is poly-ubiquitinated *via* lysine 48-linked chains in BEAS-2B cells subjected to H/R.

To clarify the relationship between the H/R with the ubiquitination of uPAR. Lysates generated from BEAS-2B cells subjected with or without H/R ± MG-132 were immunoprecipitated using an anti-uPAR antibody. As Figure 5A shows, in the presence of MG-132, H/R increases the ubiquitination level of uPAR in IP but does not affect the content of uPAR in WB. In the absence of MG-132, H/R reduces the content of uPAR. These data demonstrate that H/R upregulates the ubiquitination level of uPAR and leads to the proteasome-related degradation of uPAR. Poly-ubiquitylation can happen at multiple lysine residues, of which lysine 48 and lysine 63 linked ubiquitylation are the most common (Komander and Rape, 2012). Whereas lysine-63 linked ubiquitylation is associated with cell signaling, lysine-48 linked ubiquitylation is associated with proteasome-mediated degradation. Figure 5C revealed that lysine-48 linked poly-ubiquitylated of uPAR in BEAS-2B cells subjected to H/R. However, lysine-63-linked ubiquitylation was not detected (the data were not shown). These results thus confirmed that uPAR is poly-ubiquitylated and degraded by proteasome when cells are subjected to H/R, and the degradation was significantly weakened following treatment with exogenous uPA.

## Discussion

APE is widely treated with the serine protease UK or uPA, which functions by resolving blood clots *via* catalyzing the conversion of plasminogen to plasmin. Treatment with recombinant uPA has been shown to increase the endogenous expression of uPA and its cognate receptor, uPAR (Semina et al., 2020). The uPAR functions as the high-affinity receptor for uPA, and binding uPA to uPAR stimulates proteolytic activity on the cell membrane, in turn promoting localized plasminogen activation and degradation of the extracellular matrix. It has been previously shown that during vascular remodeling endocytosis and nuclear translocation of cell surface uPAR is mediated by SUMOylated RanGAP1 (Kiyan et al., 2012). However, to the best of our knowledge, the current study provides the first evidence that uPAR itself is degraded during simulated APE *in vitro* (hypoxia/reoxygenation), which is effectively inhibited following treatment with exogenous uPA. H/R induces apoptosis and increases secretion of TNFα, BNP, and CX3CL1, whereas treatment with UK resulted in significant inhibition of both the chymotrypsin-like (LLVY) and peptidylglutamyl peptide-hydrolyzing (LLE) peptidase activities of the proteasome. Together, these observations represent an interesting, not yet fully addressed feature of the uPA/uPAR system. Our results could have important functional implications because they point to the as yet unknown evidence that uPAR itself is degraded during simulated APE *in vitro*.

The uPA/uPAR system functions in preventing numerous pathophysiological conditions related to vascular remodeling (Kiyan et al., 2012). Vascular injury results in the induction of expression of uPA and uPAR, which then function in neointima formation during atherosclerosis (Steins et al., 2004). The uPA/uPAR system functions in endothelial cell migration and proliferation, fibrin deposition, wound healing, fibroblast to myofibroblast differentiation, and epithelial to mesenchymal transition (Steins et al., 2004; Gu et al., 2005; Shushakova et al., 2005; Bernstein et al., 2007; Binder et al., 2007; Kiyan et al., 2009; Kiyan et al., 2012). Interaction of uPAR with different proteins potentiates its function in regulating downstream signaling pathways functioning in cell adhesion, migration, proliferation, inflammation, and apoptosis (Blasi and Carmeliet, 2002). APE is also associated with inflammatory response and part of the protective action of exogenous uPA might be due to the ability to uPAR to inhibit signaling pathways associated with inflammation and cell death (Zagorski et al., 2003; Zhang et al., 2007; Wang et al., 2009; Apostolakis and Spandidos, 2013; Wang et al., 2013; Wang et al., 2014; Zhang et al., 2017; Shi et al., 2018), in turn alleviating APE-associated ischemia and pulmonary hypertension. Given the importance of the uPA/uPAR axis in a multitude of pathophysiological contexts, our results provide important yet undefined mechanistic insights.

The results of the present study indicated that global activation of 20S proteolytic activity following induction of H/R. It will be important to determine in future studies the identity of proteins that are degraded following H/R or APE and the degradation of what portion of those proteins are inhibited *via* UK treatment. In addition, the E3 ligase responsible for the degradation of uPAR remains to be determined. It will be interesting to identify if multiple E3 ligases are activated or if a single E3 ligase is activated following APE or H/R. E3 ligases are highly druggable and hence identification of the E3 ligase(s) activated following APE might provide more potent alternative therapeutic targets that alone or in combination with UK will have synergistic outcomes in patients with APE.

The present study has certain limitations since we used only one bronchial epithelial BEAS-2B cell. Therefore, the same mechanisms of uPAR in other cells need further investigation. In addition, it was based on an *in vitro* H/R model that simulates APE but was definitely not APE. Hence, whether similar mechanisms are functioning in *in vivo* models of APE remains to be investigated in future studies. In conclusion, our study reveals that H/R induces ubiquitylation and proteasome-mediated degradation of uPAR, which is attenuated when cells are treated with exogenous uPA. Given the wide functionality of the uPA/uPAR system in a vast array of pathophysiological conditions, including vascular remodeling, cardiovascular disorders, and cancer, our findings provide important mechanistic insights into these processes.

## Data Availability

The original contributions presented in the study are included in the article further inquiries can be directed to the corresponding author.

## References

[B1] Apostolakis S. Spandidos D. (2013). Chemokines and Atherosclerosis: Focus on the CX3CL1/CX3CR1 Pathway. Acta Pharmacol. Sin. 34 (10), 1251–1256. 10.1038/aps.2013.92 23974513 PMC4002164

[B2] Bdeir K. Murciano J. C. Tomaszewski J. Koniaris L. Martinez J. Cines D. B. (2000). Urokinase Mediates Fibrinolysis in the Pulmonary Microvasculature. Blood 96 (5), 1820–1826. 10.1182/blood.v96.5.1820 10961882

[B3] Bernstein A. M. Twining S. S. Warejcka D. J. Tall E. Masur S. K. (2007). Urokinase Receptor Cleavage: a Crucial Step in Fibroblast-To-Myofibroblast Differentiation. Mol. Biol. Cell 18 (7), 2716–2727. 10.1091/mbc.e06-10-0912 17507651 PMC1924808

[B4] Binder B. R. Mihaly J. Prager G. W. (2007). uPAR-uPA-PAI-1 Interactions and Signaling: a Vascular Biologist's View. Thromb. Haemost. 97 (3), 336–342. 17334498

[B5] Blasi F. Carmeliet P. (2002). uPAR: a Versatile Signalling Orchestrator. Nat. Rev. Mol. Cell Biol. 3 (12), 932–943. 10.1038/nrm977 12461559

[B6] Cheng X. He J. Gao M. Chen G. Li S. Zhang Z. (2002). Multicenter Clinical Trial on the Efficacy of Thrombolytic Therapy with Urokinase And/or Anticoagulant with Low Molecular Weight Heparin in Acute Pulmonary Embolism. Zhonghua Nei Ke Za Zhi 41 (1), 6–10. 11940288

[B7] Cohen A. T. Agnelli G. Anderson F. A. Arcelus J. I. Bergqvist D. Brecht J. G. (2007). Venous Thromboembolism (VTE) in Europe. The Number of VTE Events and Associated Morbidity and Mortality. Thromb. Haemost. 98 (4), 756–764. 17938798 10.1160/TH07-03-0212

[B8] Craiu A. Gaczynska M. Akopian T. Gramm C. F. Fenteany G. Goldberg A. L. (1997). Lactacystin and Clasto-Lactacystin Beta-Lactone Modify Multiple Proteasome Beta-Subunits and Inhibit Intracellular Protein Degradation and Major Histocompatibility Complex Class I Antigen Presentation. J. Biol. Chem. 272 (20), 13437–13445. 10.1074/jbc.272.20.13437 9148969

[B9] Geerts W. H. Bergqvist D. Pineo G. F. Heit J. A. Samama C. M. Lassen M. R. (2008). Prevention of Venous Thromboembolism: American College of Chest Physicians Evidence-Based Clinical Practice Guidelines (8th Edition). Chest 133 (6 Suppl. l), 381S–453S. 10.1378/chest.08-0656 18574271

[B10] Gu J. M. Johns A. Morser J. Dole W. P. Greaves D. R. Deng G. G. (2005). Urokinase Plasminogen Activator Receptor Promotes Macrophage Infiltration into the Vascular Wall of ApoE Deficient Mice. J. Cell Physiol. 204 (1), 73–82. 10.1002/jcp.20262 15573379

[B11] Hezer H. Kiliç H. Abuzaina O. KaralezliHasanoǧlu A. (2019). Long-term Results of Low-Dose Tissue Plasminogen Activator Therapy in Acute Pulmonary Embolism. J. Investig. Med. 67, 1142–1147. 10.1136/jim-2019-001042 31341000

[B12] Hobler S. C. Williams A. Fischer D. Wang J. J. Sun X. Fischer J. E. (1999). Activity and Expression of the 20S Proteasome Are Increased in Skeletal Muscle during Sepsis. Am. J. Physiol. 277 (2), R434–R440. 10.1152/ajpregu.1999.277.2.R434 10444550

[B13] Kiyan J. Smith G. Haller H. Dumler I. (2009). Urokinase-receptor-mediated Phenotypic Changes in Vascular Smooth Muscle Cells Require the Involvement of Membrane Rafts. Biochem. J. 423 (3), 343–351. 10.1042/BJ20090447 19691446

[B14] Kiyan Y. Limbourg A. Kiyan R. Tkachuk S. Limbourg F. P. Ovsianikov A. (2012). Urokinase Receptor Associates with Myocardin to Control Vascular Smooth Muscle Cells Phenotype in Vascular Disease. Arterioscler. Thromb. Vasc. Biol. 32 (1), 110–122. 10.1161/ATVBAHA.111.234369 22075245

[B15] Komander D. Rape M. (2012). The Ubiquitin Code. Annu. Rev. Biochem. 81, 203–229. 10.1146/annurev-biochem-060310-170328 22524316

[B16] Lang I. M. Pesavento R. Bonderman D. Yuan J. X. (2013). Risk Factors and Basic Mechanisms of Chronic Thromboembolic Pulmonary Hypertension: a Current Understanding. Eur. Respir. J. 41 (2), 462–468. 10.1183/09031936.00049312 22700839

[B17] Leth J. M. Leth-Espensen K. Z. Kristensen K. K. Kumari A. Lund Winther A. M. Young S. G. (2019). Evolution and Medical Significance of LU Domain-Containing Proteins. Int. J. Mol. Sci. 20. 10.3390/ijms20112760 PMC6600238 31195646

[B18] Liu Y. Wang C. Yang Y. Hou X. Wang J. (2008). Pro-urokinase Up-Regulates the Expression of Urokinase-type Plasminogen Activator (u-PA) in Human Pulmonary Arterial Endothelial Cells. Thromb. Res. 121 (4), 485–491. 10.1016/j.thromres.2007.05.021 17640719

[B19] Ploug M. Rønne E. Behrendt N. Jensen A. L. Blasi F. Danø K. (1991). Cellular Receptor for Urokinase Plasminogen Activator. Carboxyl-Terminal Processing and Membrane Anchoring by Glycosyl-Phosphatidylinositol. J. Biol. Chem. 266, 1926–1933. 10.1016/s0021-9258(18)52382-6 1846368

[B20] Saghazadeh A. Hafizi S. Rezaei N. (2015). Inflammation in Venous Thromboembolism: Cause or Consequence? Int. Immunopharmacol. 28 (1), 655–665. 10.1016/j.intimp.2015.07.044 26253657

[B21] Saghazadeh A. Rezaei N. (2016). Inflammation as a Cause of Venous Thromboembolism. Crit. Rev. Oncol. Hematol. 99, 272–285. 10.1016/j.critrevonc.2016.01.007 26811138

[B22] Sasahara A. A. Cannilla J. E. Belko J. S. Morse R. L. Criss A. J. (1967). Urokinase Therapy in Clinical Pulmonary Embolism. A New Thrombolytic Agent. N. Engl. J. Med. 277 (22), 1168–1173. 10.1056/NEJM196711302772203 6058604

[B23] Sasaki K. Moriyama S. Tanaka Y. Sumi H. Toki N. Robbins K. C. (1985). The Transport of 125I-Labeled Human High Molecular Weight Urokinase across the Intestinal Tract in a Dog Model with Stimulation of Synthesis And/or Release of Plasminogen Activators. Blood 66 (1), 69–75. 10.1182/blood.v66.1.69.bloodjournal66169 4039958

[B24] Semina E. V. Rubina K. A. Shmakova A. A. Rysenkova K. D. Klimovich P. S. Aleksanrushkina N. A. (2020). Downregulation of uPAR Promotes Urokinase Translocation into the Nucleus and Epithelial to Mesenchymal Transition in Neuroblastoma. J. Cell Physiol. 235, 6268–6286. 10.1002/jcp.29555 31990070 PMC7318179

[B25] Shi Y. Zhang Z. Cai D. Kuang J. Jin S. Zhu C. (2018). Urokinase Attenuates Pulmonary Thromboembolism in an Animal Model by Inhibition of Inflammatory Response. J. Immunol. Res. 2018, 6941368. 10.1155/2018/6941368 30671487 PMC6323506

[B26] Shushakova N. Eden G. Dangers M. Zwirner J. Menne J. Gueler F. (2005). The Urokinase/urokinase Receptor System Mediates the IgG Immune Complex-Induced Inflammation in Lung. J. Immunol. 175 (6), 4060–4068. 10.4049/jimmunol.175.6.4060 16148155

[B27] Steins M. B. Padró T. Schwaenen C. Ruiz S. Mesters R. M. Berdel W. E. (2004). Overexpression of Urokinase Receptor and Cell Surface Urokinase-type Plasminogen Activator in the Human Vessel Wall with Different Types of Atherosclerotic Lesions. Blood Coagul. Fibrinolysis 15 (5), 383–391. 10.1097/01.mbc.0000114441.59147.56 15205586

[B28] Sumi H. Seiki M. Morimoto N. Tsushima H. Maruyama M. Mihara H. (1985). Plasma Fibrinolysis after Intraduodenal Administration of Urokinase in Rats. Enzyme 33 (3), 122–127. 10.1159/000469420 4054071

[B29] Toki N. Sumi H. Sasaki K. Boreisha I. Robbins K. C. (1985). Transport of Urokinase across the Intestinal Tract of Normal Human Subjects with Stimulation of Synthesis And/or Release of Urokinase-type Proteins. J. Clin. Invest. 75 (4), 1212–1222. 10.1172/JCI111818 3921568 PMC425447

[B30] Wang C. Zhai Z. Yang Y. Yuan Y. Cheng Z. Liang L. (2009). Efficacy and Safety of 2-hour Urokinase Regime in Acute Pulmonary Embolism: a Randomized Controlled Trial. Respir. Res. 10, 128. 10.1186/1465-9921-10-128 20040086 PMC2806365

[B31] Wang L. Wu J. Zhang W. Zhi Y. Wu Y. Jiang R. (2013). Effects of Aspirin on the ERK and PI3K/Akt Signaling Pathways in Rats with Acute Pulmonary Embolism. Mol. Med. Rep. 8 (5), 1465–1471. 10.3892/mmr.2013.1676 24026640

[B32] Wang L. C. Jiang R. L. Zhang W. Wei L. L. Yang R. H. (2014). Effects of Aspirin on the Expression of Nuclear Factor-Κb in a Rat Model of Acute Pulmonary Embolism. World J. Emerg. Med. 5 (3), 229–233. 10.5847/wjem.j.issn.1920-8642.2014.03.013 25225590 PMC4163806

[B33] Xu D. Bum‐Erdene K. Leth J. M. Ghozayel M. K. Ploug M. Meroueh S. O. (2020). Small‐Molecule Inhibition of the uPAR ⋅ uPA Interaction by Conformational Selection. ChemMedChem 16, 377–387. 10.1002/cmdc.202000558 33107192

[B34] Yang Y. Liang L. Zhai Z. He H. Xie W. Peng X. (2011). Pulmonary Embolism Incidence and Fatality Trends in Chinese Hospitals from 1997 to 2008: a Multicenter Registration Study. PLoS One 6 (11), e26861. 10.1371/journal.pone.0026861 22069474 PMC3206059

[B35] Zagorski J. Debelak J. Gellar M. Watts J. A. Kline J. A. (2003). Chemokines Accumulate in the Lungs of Rats with Severe Pulmonary Embolism Induced by Polystyrene Microspheres. J. Immunol. 171 (10), 5529–5536. 10.4049/jimmunol.171.10.5529 14607960

[B36] Zhang Y. Ma L. Fu Q. Zhao T. Yan R. Y. Su X. (2019). Comparison of Urokinase and Reteplase Thrombolytic Treatment in Patients with High-Risk Pulmonary Embolism. Exp. Ther. Med. 18 (6), 4804–4810. 10.3892/etm.2019.8153 31798706 PMC6880444

[B37] Zhang Y. Sun T. He B. Wang L. (2007). Thrombolytic Therapy with Urokinase for Pulmonary Embolism in Patients with Stable Hemodynamics. Med. Sci. Monit. 13 (1), CR20–3. 17179905

[B38] Zhang Z. Yang W. Ying R. Shi Y. Jiang H. Cai D. (2017). Influence of Aspirin on the CX3CL1/CX3CR1 Signaling Pathway in Acute Pulmonary Embolism. Int. J. Mol. Med. 39 (6), 1580–1588. 10.3892/ijmm.2017.2969 28487961

[B39] Zhao T. Ni J. Hu X. Wang Y. Du X. (2018). The Efficacy and Safety of Intermittent Low-Dose Urokinase Thrombolysis for the Treatment of Senile Acute Intermediate-High-Risk Pulmonary Embolism: A Pilot Trial. Clin. Appl. Thromb. Hemost. 24 (7), 1067–1072. 10.1177/1076029618758953 29552916 PMC6714758

